# Supplementary material for: Full-duplex reflective beamsteering metasurface featuring magnetless nonreciprocal amplification
Source: Nat Commun. 2021 Jul 20;12:4414. doi: 10.1038/s41467-021-24749-7 (PMC8292412; doi:10.1038/s41467-021-24749-7)
Supplement: Supplementary file 1 — Supplementary Information for "Full-Duplex Re ective Beamsteering Metasurface Featuring Magnetless Nonreciprocal Amplication" [file 41467_2021_24749_MOESM1_ESM.pdf]

Supplementary Information for "Full-Duplex Reflective Beamsteering Metasurface Featuring Magnetless Nonreciprocal Amplification"

Sajjad Taravati and George V. Eleftheriades
Department of Electrical and Computer Engineering,
University of Toronto, Toronto,
Ontario M5S 2E4, Canada
email: sajjad.taravati@utoronto.ca

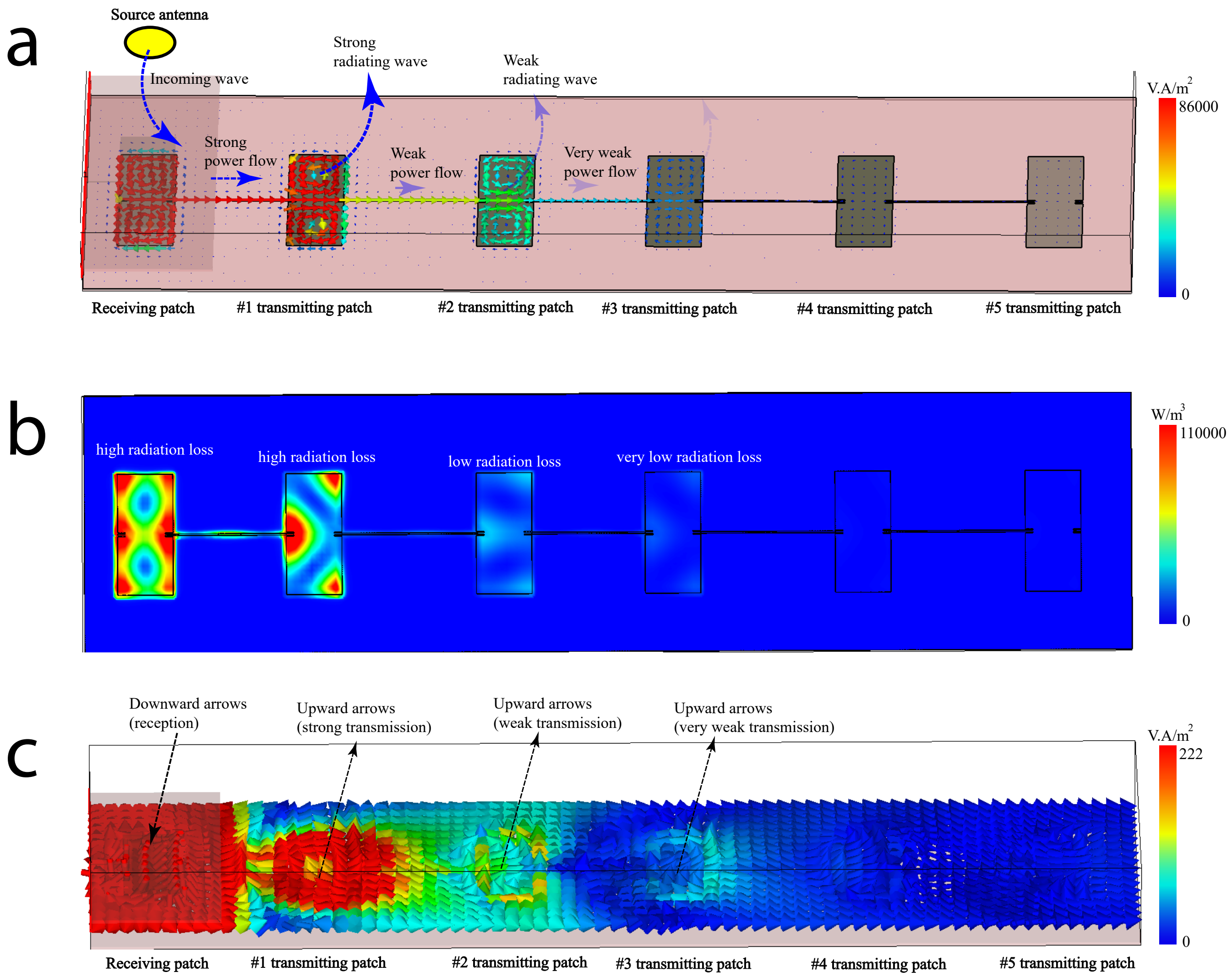

Supplementary Fig. 1. Simulation results for a chain formed by six interconnected patch radiators. **a** Power flow inside the interconnected chain structure. **b** Power loss inside the chain. **c** Power flow on top of the interconnected chain structure.

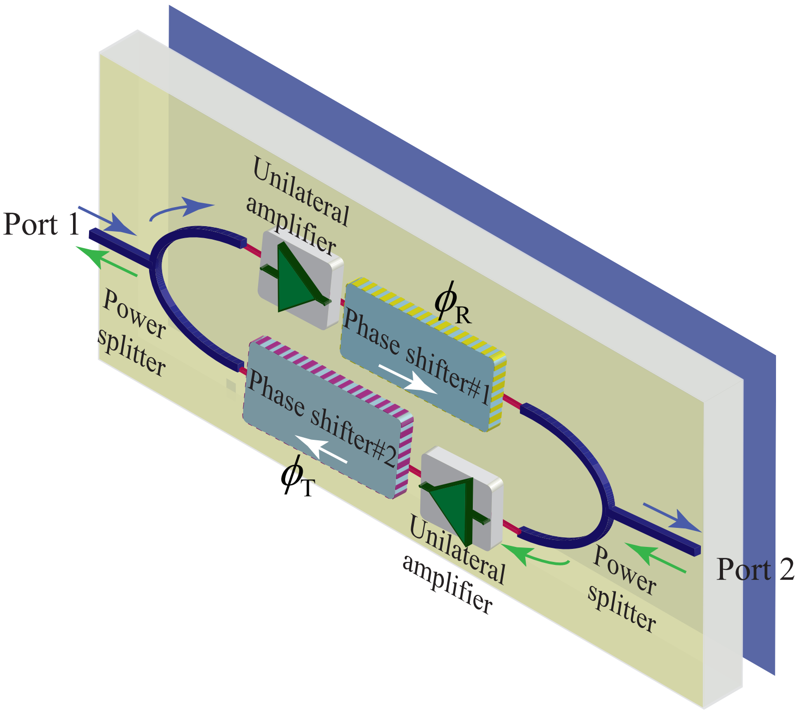

Supplementary Fig. 2. Bidirectional nonreciprocal phase shifter and amplifier composed of two amplifiers, two reciprocal phase shifters, and two power splitters.
